# Proinflammatory cytokines and lipopolysaccharides up regulate MMP-3 and MMP-13 production in Asian elephant (*Elephas maximus*) chondrocytes: attenuation by anti-arthritic agents

**DOI:** 10.1186/s12917-019-2170-8

**Published:** 2019-11-21

**Authors:** Nutnicha Sirikaew, Siriwadee Chomdej, Siriwan Tangyuenyong, Weerapongse Tangjitjaroen, Chaleamchat Somgird, Chatchote Thitaram, Siriwan Ongchai

**Affiliations:** 1 Thailand Excellence Center for Tissue Engineering and Stem Cells, Department of Biochemistry, Faculty of Medicine, Chiang Mai University, 110 Intrawarorot Rd., Chiang Mai, 50200 Thailand; 2 Department of Biology, Faculty of Science, Chiang Mai University, Chiang Mai, 50200 Thailand; 3 Department of Companion Animal and Wildlife Clinic, Faculty of Veterinary Medicine, Chiang Mai University, Chiang Mai, 50100 Thailand

**Keywords:** *Elephas maximus*, Osteoarthritis, Proinflammatory cytokines, MMP-3, MMP-13

## Abstract

**Background:**

Osteoarthritis (OA), the most common form of arthritic disease, results from destruction of joint cartilage and underlying bone. It affects animals, including Asian elephants (*Elephas maximus*) in captivity, leading to joint pain and lameness. However, publications regarding OA pathogenesis in this animal are still limited. Therefore, this study aimed to investigate the effect of proinflammatory cytokines, including interleukin-1 beta (IL-1β), IL-17A, tumor necrosis factor-alpha (TNF-α), and oncostatin M (OSM), known mediators of OA pathogenesis, and lipopolysaccharides on the expression of cartilaginous degrading enzymes, matrix metalloproteinase (MMP)-3 and MMP-13, in elephant articular chondrocytes (ELACs) cultures. Anti-arthritic drugs and the active compounds of herbal plants were tested for their potential attenuation against overproduction of these enzymes.

**Results:**

Among the used cytokines, OSM showed the highest activation of *MMP3* and *MMP13* expression, especially when combined with IL-1β. The combination of IL-1β and OSM was found to activate phosphorylation of the mitogen-activated protein kinase (MAPK) pathway in ELACs. Lipopolysaccharides or cytokine-induced expressions were suppressed by pharmacologic agents used to treat OA, including dexamethasone, indomethacin, etoricoxib, and diacerein, and by three natural compounds, sesamin, andrographolide, and vanillylacetone.

**Conclusions:**

Our results revealed the cellular mechanisms underlying OA in elephant chondrocytes, which is triggered by proinflammatory cytokines or lipopolysaccharides and suppressed by common pharmacological or natural medications used to treat human OA. These results provide a more basic understanding of the pathogenesis of elephant OA, which could be useful for adequate medical treatment of OA in this animal.

## Background

Osteoarthritis (OA), the most prevalent arthritic disease, is characterized by cartilage degradation and consequent joint pain and disability [1, 2]. OA affects many species, including elephants, especially Asian elephants (*Elephas maximus*) kept in captivity. Excessive body weight along with the captive environment and trained behaviors are critical factors of OA pathogenesis in elephants [3, 4]. These factors disturb the equilibrium between the synthesis and degradation of the extracellular matrix (ECM) by chondrocytes, leading to further degradation of the ECM by matrix-degrading enzymes, especially matrix metalloproteinases (MMPs) [5]. The disturbance of this equilibrium is found particularly among captive elephants [6].

MMPs are a group of zinc-dependent endopeptidases that, when in excess, cause degeneration of the cartilage ECM. There has been a reported increase in MMP-3 and MMP-13 in humans and animals with OA, suggesting that these MMPs play a pivotal role in OA cartilage destruction [7–10]. It has previously been shown that the production of matrix-degrading enzymes is activated by proinflammatory cytokines, including interleukin-1 beta (IL-1β), IL-17A, tumor necrosis factor-alpha (TNF-α), and oncostatin M (OSM) [11–14]. In addition, the combination of OSM with other proinflammatory cytokines causes the greatest loss of cartilage matrix in OA [15–17]. Moreover, lipopolysaccharides (LPS), i.e., outer-membrane components of Gram-negative bacteria, contribute to septic arthritis and cartilage degeneration by upregulating the synthesis of catabolic factors, including proinflammatory cytokines and matrix-degrading enzymes [18, 19]. In OA pathogenesis, cytokine-induced signal transduction involves the activation of several pathways, including those of the mitogen-activated protein kinase (MAPK) family [20].

OA in elephants is caused by an imbalance of pressure on joints, which in turn is caused by a lack of exercise or an excessive body weight. This damages the cartilage, releasing inflammatory mediators and enzymes and, consequently, leading to joint inflammation. Affected elephants show signs of lameness and joint swelling and are reluctant to lay down because it will be difficult to stand up again. Swimming in a big pool to reduce weight bearing and administration of anti-inflammatory drugs are considered suitable treatments [21].

Current pharmacologic approaches for OA treatment aim at reducing inflammation and pain, improving joint function, and delaying disease progression. Commonly used medicines include steroids, non-steroidal anti-inflammatory drugs (NSAIDs), and disease-modifying OA drugs (DMOADs) [22], among which the most common agents are dexamethasone, indomethacin, etoricoxib, and diacerein, which have been shown to inhibit the expression of MMPs such as *MMP1*, *MMP2*, *MMP3*, *MMP9*, and *MMP13* [23–26]. However, these substances are associated with a high incidence of adverse effects, including gastrointestinal damage and heart failure [27]. Thus, natural product-derived compounds with anti-inflammatory activity and low toxicity have become alternative treatments for OA. Among such compounds, sesamin, andrographolide, and vanillylacetone or zingerone have been reported to exhibit chondroprotective activity by inhibiting the expression of *MMP1*, *MMP3*, and *MMP13* in chondrocytes [28–30].

It was reported that IL-1β stimulated the degradation of elephant cartilage in an explant culture model [31]. However, the existence of published studies on the cellular mechanisms of OA in elephants is limited. Therefore, the present study aimed to investigate the molecular mechanisms underlying the activation of expression of MMP-3 and MMP-13 by proinflammatory cytokines and LPS in elephant articular chondrocytes (ELACs). Additionally, the ability of commonly used anti-OA medications and natural compounds to inhibit these mechanisms was investigated. The information gained from this study will be useful in improving the treatment of elephants with OA and in supporting further research on elephant degenerative arthritis, both of which are important for a better quality of life for the elephants and contribute to vital elephant conservation.

## Results

### Proinflammatory cytokines induced upregulation of *MMP3* and *MMP13* expression in ELACs culture

**Fig. 1:**
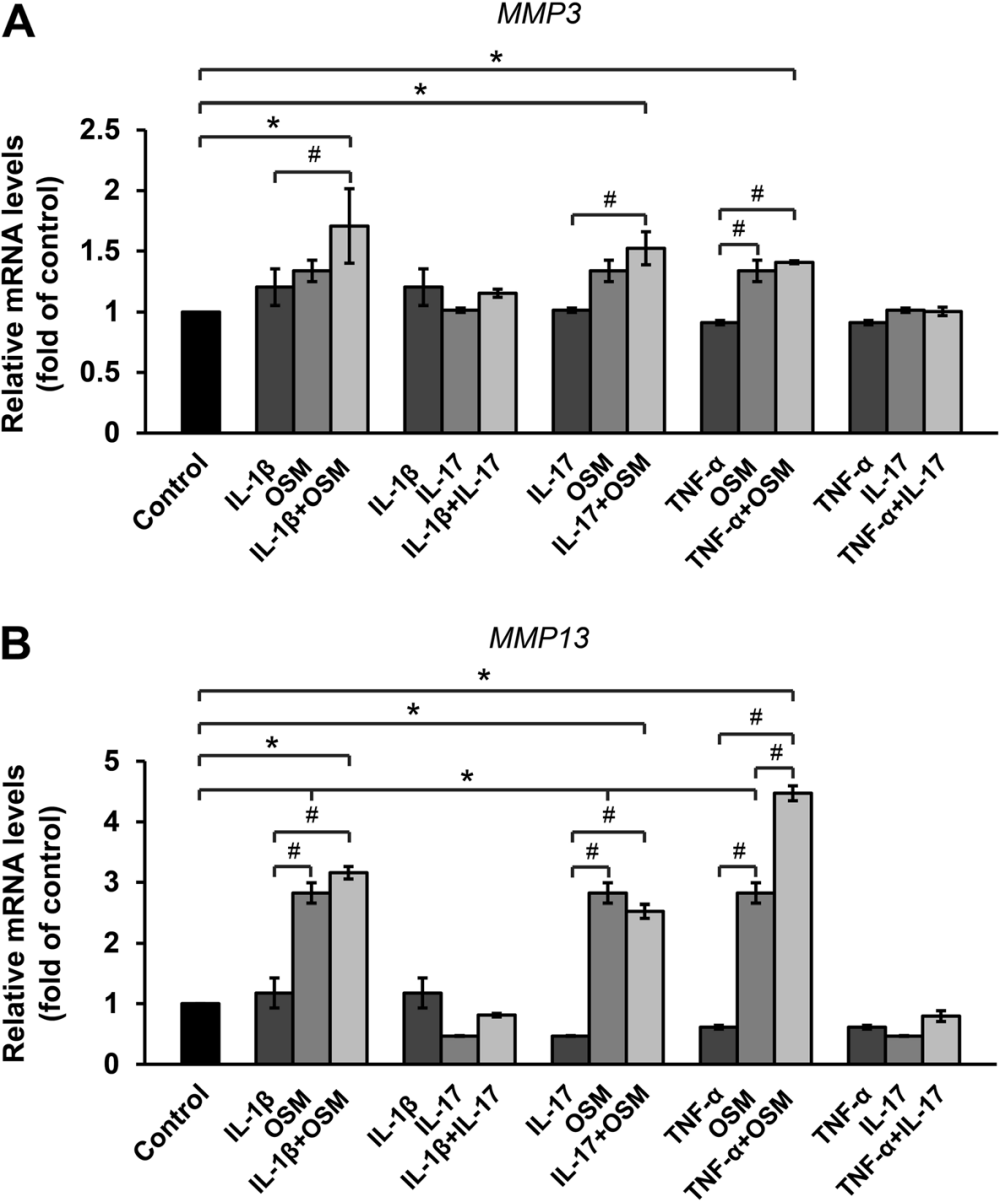
Proinflammatory cytokines upregulate the mRNA expression of *MMP3* (**a**) and *MMP13* (**b**) in ELACs. The chondrocytes were treated with individual proinflammatory cytokines as follows: IL-1β (2.5 ng/mL); IL-17A (5 ng/mL); and TNF-α (5 ng/mL), or their combination with OSM (2 ng/mL) or IL-17A (5 ng/mL), for 24 h. mRNA levels were assessed by real-time RT-PCR. Results are presented as mean ± SEM. * signifies statistical significance compared with control (*p < 0.05), whereas # signifies statistical significance in relation to single-cytokine treatment (#*p* < 0.05)

**Fig. 2:**
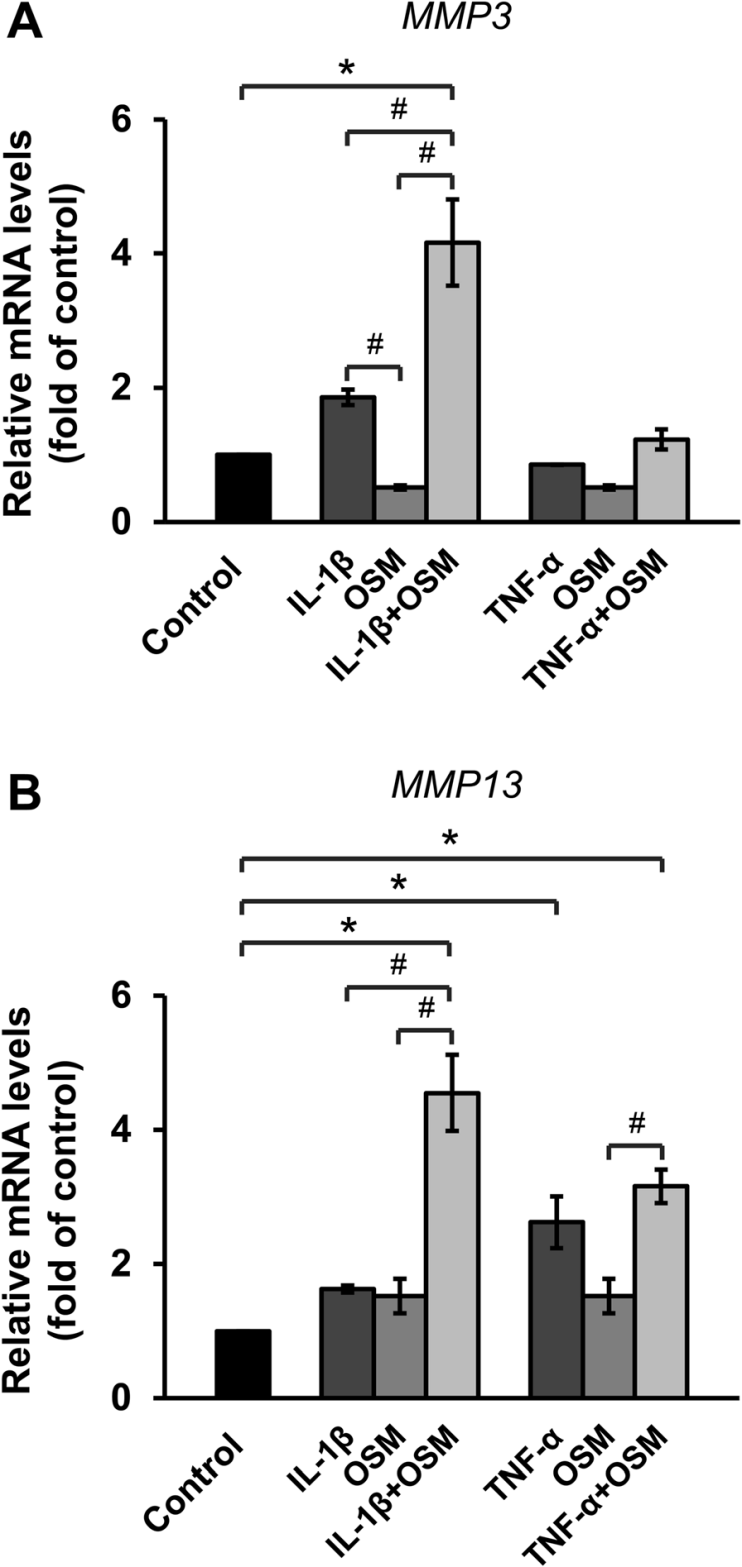
IL-1β in combination with OSM stimulates expression of *MMP3* (**a**) and *MMP13* (**b**) in ELAC pellets culture. ELAC pellets were treated with IL-1β or TNF-α, alone or in combination with OSM, for 3 days. The mRNA levels were assessed by real-time RT-PCR. Results are presented as mean ± SEM. * signifies statistical significance compared with control (*p < 0.05), whereas # signifies statistical significance in relation to single-cytokine treatment (#*p* < 0.05)

Treatment with OSM alone resulted in a slight increase in *MMP3* mRNA levels and a marked elevation of *MMP13* levels. However, IL-1β, IL-17A, and TNF-α did not influence the expression of these genes in the monolayer culture model (Fig. 1). The combination of cytokines OSM and TNF-α significantly induced *MMP13* expression, whereas the combination of OSM and IL-1β or IL-17A tended to induce *MMP3* expression. In the pellet culture model (Fig. 2), the results of individual cytokine treatments show that only TNF-α could significantly activate the expression of *MMP13*. Meanwhile, the results of treatments with combined cytokines demonstrate that OSM combined with IL-1β dramatically increased the expression of both *MMP3* and *MMP13*, whereas OSM combined with TNF-α slightly induced the expression of *MMP13* but not that of *MMP3*.

### Drugs and active compounds of medicinal plants inhibited cytokine-induced expression of *MMP3* and *MMP13* in ELACs culture

**Fig. 3:**
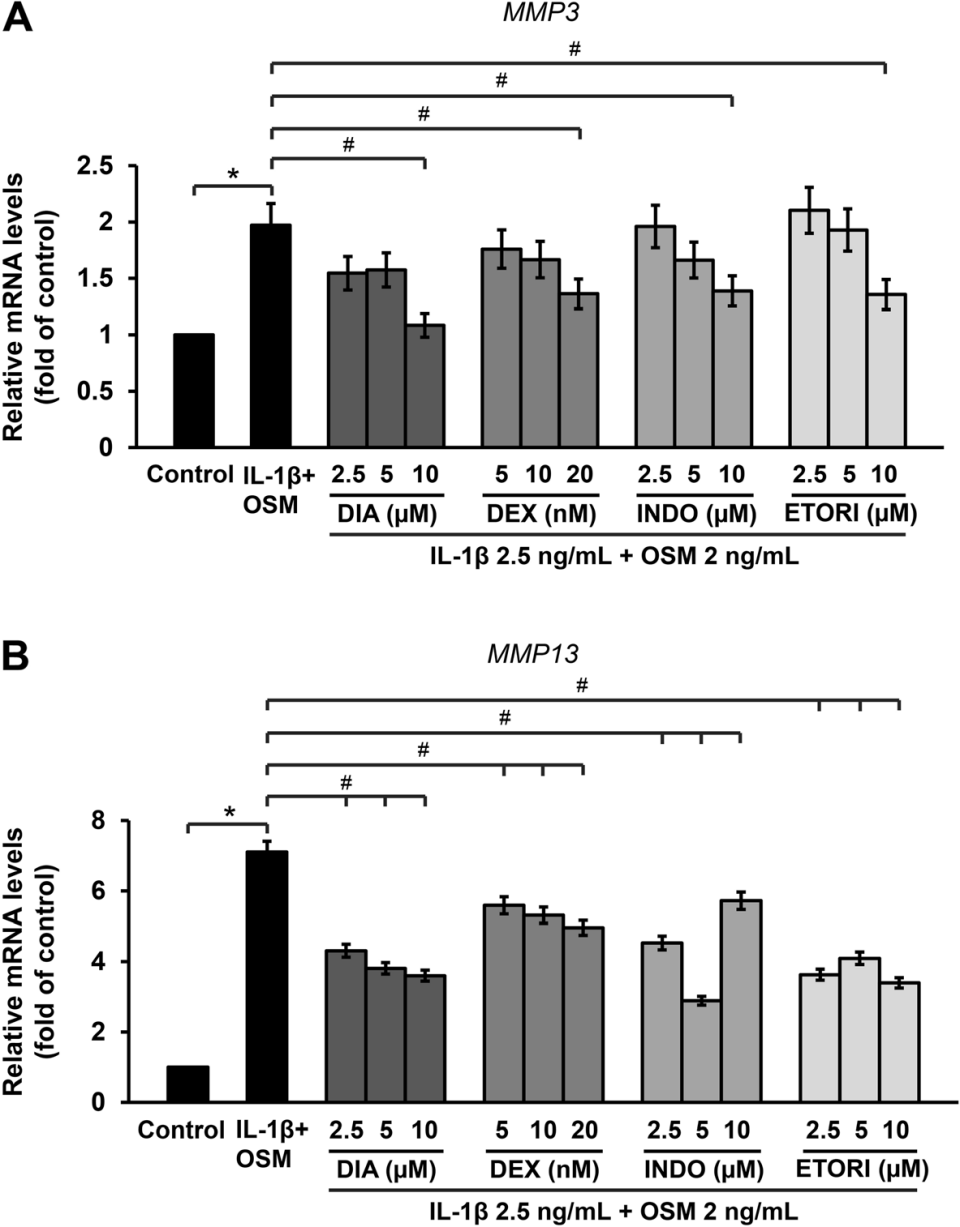
Anti-arthritic drugs decrease the cytokines-induced expressions of *MMP3* (**a**) and *MMP13* (**b**) in ELACs. Chondrocytes were pre-treated with a combination of IL-1β (2.5 ng/mL) and OSM (2 ng/mL) for 2 h, after which they were treated with various concentrations of DIA (diacerein; 2.5–10 μM), DEX (dexamethasone; 5–20 nM), INDO (indomethacin; 2.5–10 μM), and ETORI (etoricoxib; 2.5–10 μM), for 24 h. mRNA levels were assessed by real-time RT-PCR. Results are presented as mean ± SEM. * signifies statistical significance compared with control (*p < 0.05), whereas # signifies statistical significance in relation to the cytokines treatment group (#*p* < 0.05)

**Fig. 4:**
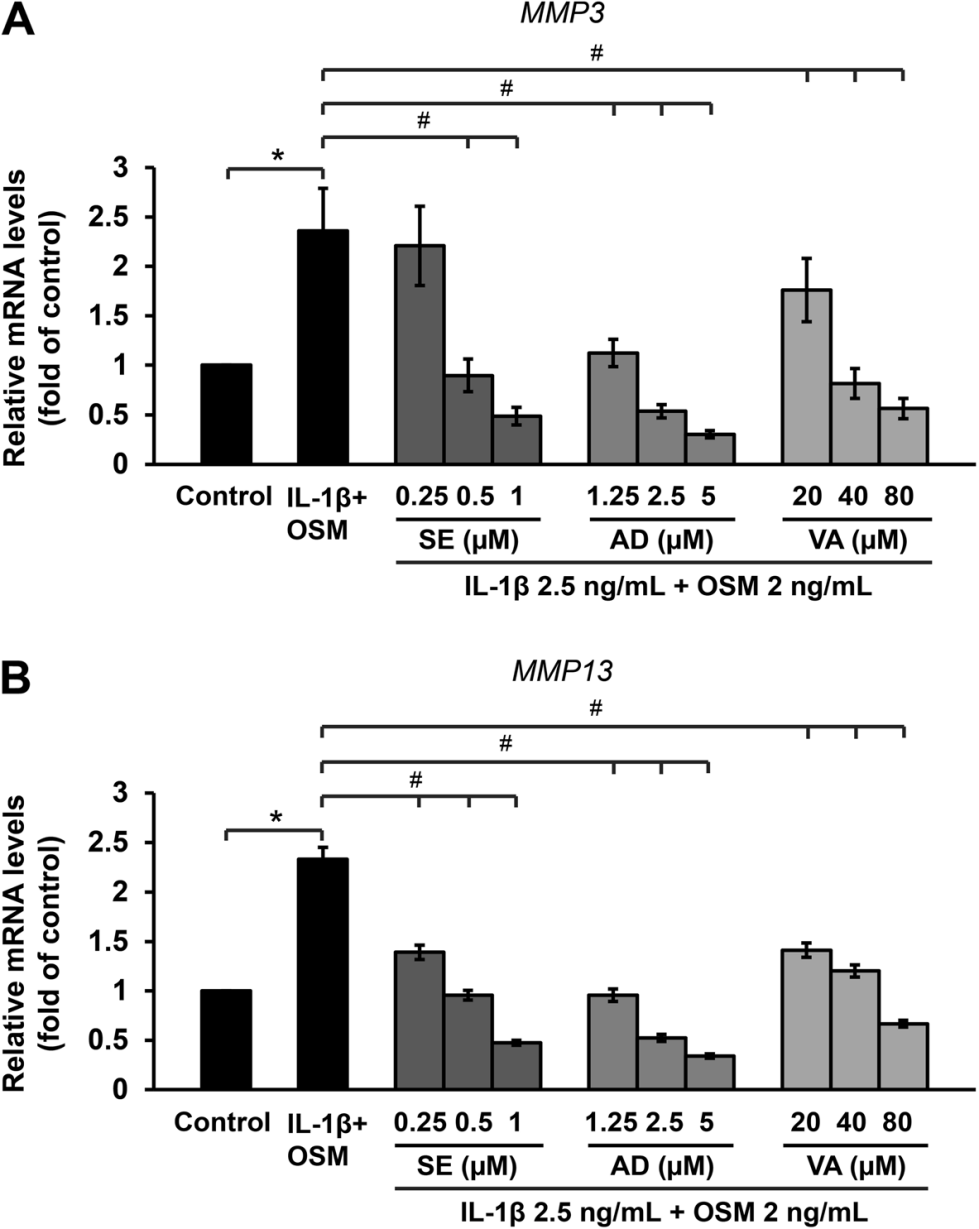
Natural active compounds reduce the cytokines-induced mRNA levels *MMP3* (**a**) and *MMP13* (**b**) in ELACs. The chondrocytes were pre-treated with a combination of IL-1β (2.5 ng/mL) and OSM (2 ng/mL) for 2 h, after which they were treated with various concentrations of SE (sesamin; 0.25–1 μM), AD (andrographolide; 1.25–5 μM), and VA (vanillylacetone; 20–80 μM), for 24 h. The mRNA levels were assessed by real-time RT-PCR. Results are presented as mean ± SEM. * signifies statistical significance compared with control (*p < 0.05), whereas # signifies statistical significance in relation to the cytokines treatment group (#*p* < 0.05)

The results show that medications used to treat OA in humans, such as diacerein, dexamethasone, indomethacin, and etoricoxib, significantly attenuated *MMP3* and *MMP13* mRNA levels in the ELACs culture (Fig. 3a and b). Likewise, natural active compounds, including sesamin, andrographolide, and vanillylacetone, significantly suppressed the *MMP3* and *MMP13* mRNA levels in a dose-dependent manner (Fig. 4a and b).

### LPS induced the expression of *MMP3* and *MMP13* along with proinflammatory cytokine genes in ELACs culture

**Fig. 5:**
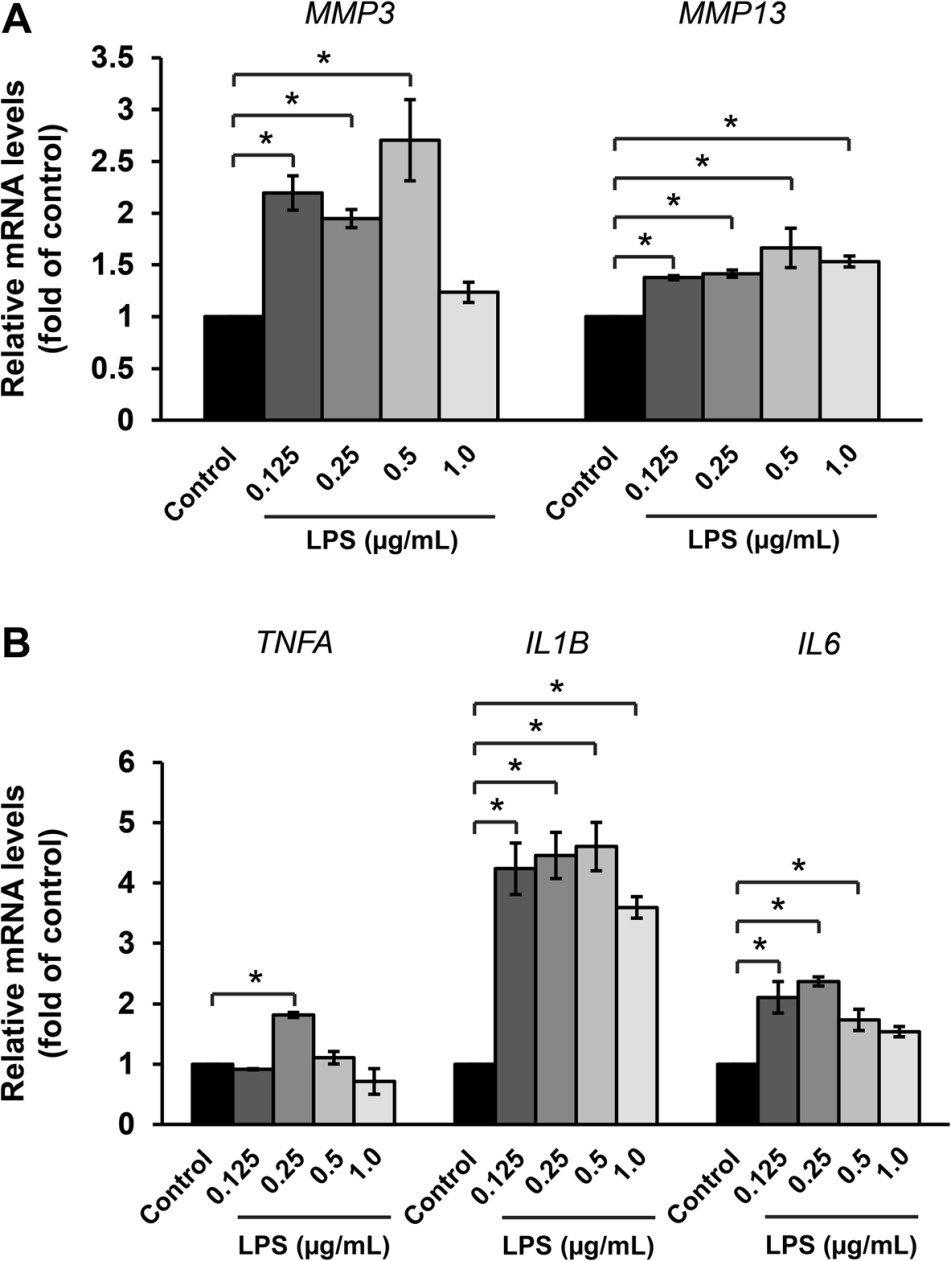
LPS induces expression of *MMP3* and *MMP13* (**a**), and proinflammatory cytokines (**b**) in ELACs culture. The chondrocytes were treated with LPS at various concentrations (0.125–1 μg/mL) for 24 h, then mRNA levels were assessed by real-time RT-PCR. Results are presented as mean ± SEM. * signifies statistical significance compared with control (*p < 0.05)

The results show that LPS at a 0.125 μg/mL concentration significantly increased *MMP3* and *MMP13* mRNA levels as well as the levels of *IL1B* and *IL6* while increasing the expression of the TNF-α gene (*TNFA*) at a concentration of only 0.25 μg/mL (Fig. 5).

**Fig. 6:**
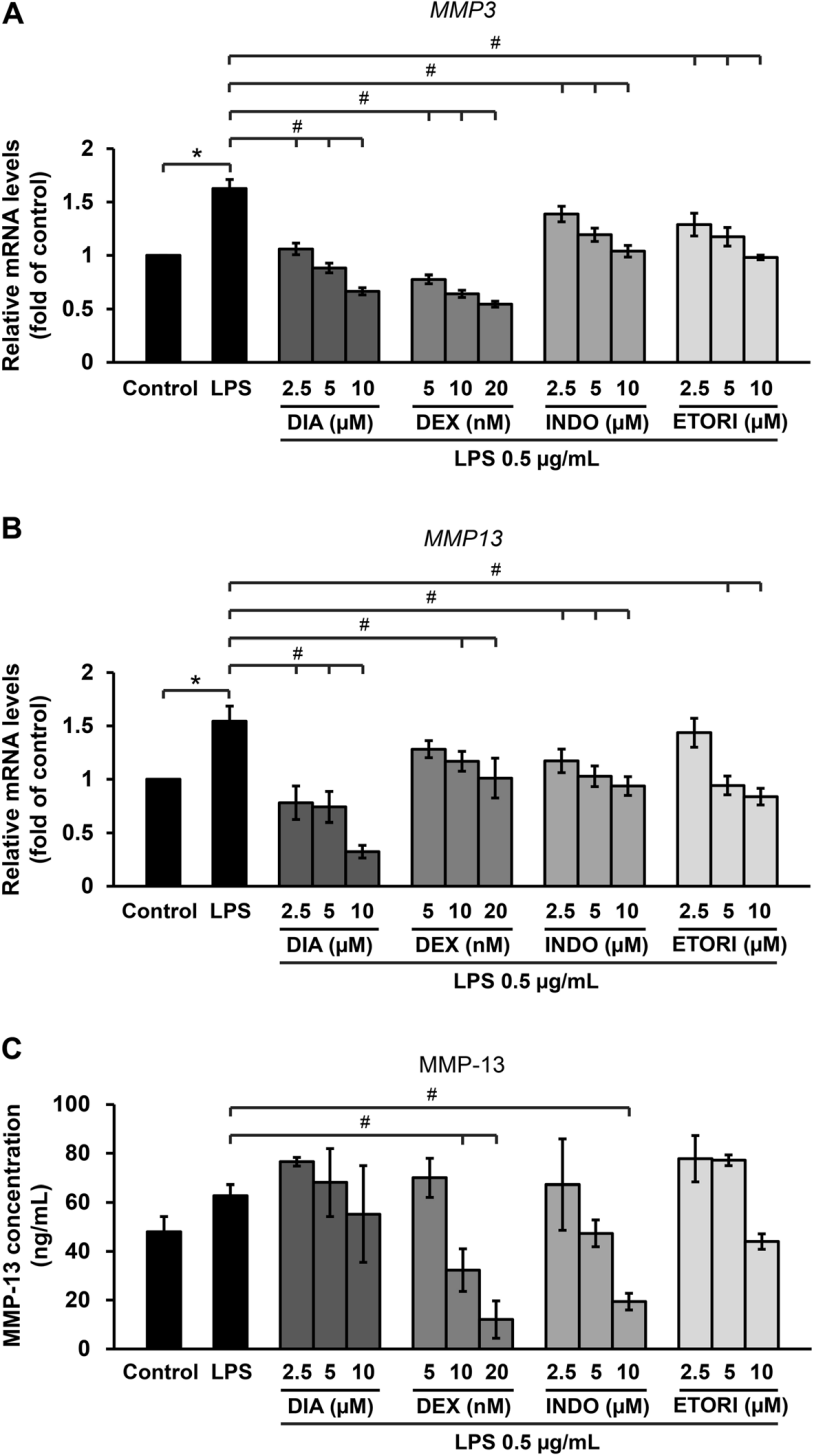
Anti-arthritic drugs suppressed mRNA levels of *MMP3* (**a**) and *MMP13* (**b**) and decreasing MMP13 protein levels (**c**). The chondrocytes were pre-treated with 0.5 μg/mL LPS for 2 h, after which they were treated with various concentrations of DIA (diacerein; 2.5–10 μM), DEX (dexamethasone; 5–20 nM), INDO (indomethacin; 2.5–10 μM), and ETORI (etoricoxib; 2.5–10 μM) for 24 h. mRNA levels were then assessed by real-time RT-PCR. Results are presented as mean ± SEM. * signifies statistical significance compared with control (*p < 0.05), whereas # signifies statistical significance in relation to the cytokines treatment group (#*p* < 0.05)

Co-treatment with LPS and anti-arthritic drugs such as diacerein, dexamethasone, indomethacin, and etoricoxib significantly suppressed *MMP3* and *MMP13* mRNA levels in a dose-dependent manner (Fig. 6a and b). Figure 6c illustrates the LPS-induced increase of MMP-13 protein levels in the culture media, which was significantly suppressed by dexamethasone and indomethacin. However, the level of MMP-3 in the culture media could not be assessed using a human MMP-3 CLIA kit (data not shown).

### Activation of the MAPK pathway in ELACs by IL-1β combined with OSM

**Fig. 7:**
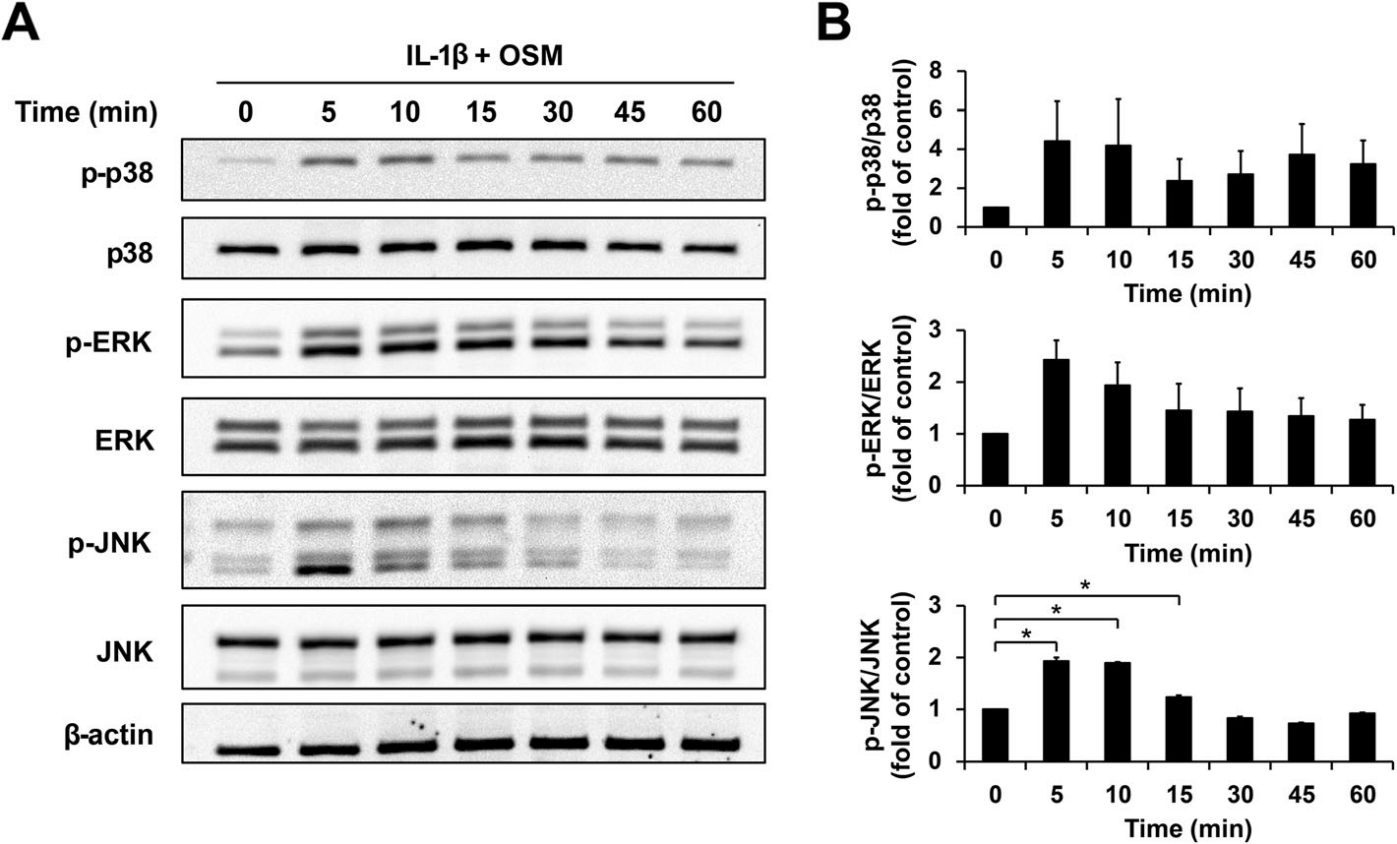
Activation of the MAPK pathway in ELACs by IL-1β combined with OSM. ELACs were stimulated by the combination of IL-1β (2.5 ng/mL) and OSM (2.5 ng/mL) at the indicated time points. Cell lysates were immunoblotted to investigate the total and phosphorylated molecular forms, which indicated an active MAPK pathway. Immunoblots are represented in (**a**) and bar graphs (**b**) show the proportion between the band intensities of phosphorylated p38, ERK, and JNK over their total forms. Results are presented as mean ± SEM. * signifies statistical significance compared with control (*p < 0.05)

The MAPK pathway, one of the molecular mechanisms involved in OA pathogenesis, was activated in ELACs treated with a combination of IL-1β and OSM. The results show that the combined proinflammatory cytokines activated the maximum phosphorylation of p38, ERK, and JNK from 5 to 10 min, followed by its gradual decrease after 15 min (Fig. 7).

## Discussion

OA is the most prevalent musculoskeletal disorder in both humans and animals. Most studies on OA have focused on humans, with few reports available on animals, especially elephants. Asian elephants kept in captivity frequently suffer from OA caused primarily by residing in damp buildings and being overworked by humans as well as by restricted movement, which leads to cartilage degeneration and lameness [3, 4]. Reports on the mechanisms underlying OA in elephants are rare.

The present study used monolayer and pellet cultures of elephant chondrocytes as in vitro models to investigate the mechanisms underlying OA pathogenesis. Although the pellet culture, a three-dimensional culture model, mimicked the chondrocytes’ microenvironment within cartilage tissue more accurately [32], two-dimensional monolayer cultures are a faster and simpler model for cell-based studies. They allowed for quick evaluation of the effects of several proinflammatory cytokines known to be involved in OA pathogenesis on the expressions of *MMP3* and *MMP13* in ELACs.

The present results clearly demonstrate that ELACs are sensitive to activation by proinflammatory cytokines. Among the proinflammatory cytokines, the treatment with OSM alone strongly induced the expression of *MMP13* in the monolayer cultures; TNF-α, which has been previously reported to induce the expression of *MMP1*, *MMP3*, and *MMP13* in equine chondrocytes [11], caused a significant upregulation of *MMP13* in the elephant chondrocyte pellet culture. IL-17A, alone or in combination with IL-1β or TNF-α, did not alter the expression of *MMP3* or *MMP13*. The treatment with a combination of IL-17A and OSM caused a slight upregulation of *MMP3* with no effect on *MMP13*. This result is inconsistent with previous studies on human cartilage cultures, which showed that the combination of IL-17A with TNF-α and OSM synergistically upregulates the expression of enzymes MMP-1 and MMP-13 [33]. This cytokine is known to be increased in the serum of OA patients, suggesting its involvement in human OA pathogenesis [34].

Although IL-1β has been reported to play a key role in the OA pathogenesis of large animals by upregulating the expression of MMP-1, MMP-3, and MMP-13 enzymes [13, 35, 36], our results clearly demonstrate that in the elephant chondrocyte pellet culture model, this cytokine could only induce the expression of *MMP3* and *MMP13* in combination with OSM. This result is consistent with a recent report suggesting that IL-1α and IL-1β are not crucial mediators of murine OA, which may explain the lack of success of IL-1-targeted therapies for OA [37]. Nevertheless, a previous report by our team demonstrated a great loss of hyaluronan from elephant cartilage explants treated with human recombinant IL-1β, suggesting the catabolic potential of this cytokine via accelerating the processes of cleavage and release of ECM biomolecules from the affected cartilage tissue, leading to degenerative cartilage in OA [31].

OSM, which belongs to the IL-6 family, is one of the proinflammatory cytokines that contribute to inflammation and cartilage destruction in degenerative arthritis [38]. OSM induces the expression of *MMP1*, *MMP3*, and *MMP13* in bovine chondrocytes [12]. This cytokine has also been reported to synergize the action of other proinflammatory cytokines such as IL-1β, TNF-α, and IL-17A, resulting in acceleration of cartilage degeneration [15–17]. In this study, in elephant chondrocytes, the combination of OSM with IL-1β exerted the strongest induction of *MMP3* and *MMP13* expression in both the monolayer and pellet culture models, whereas the combined OSM with TNF-α only influenced the expression of *MMP13*. Our results suggest a cell-type specificity in response to the activation of cytokines. Additionally, all cytokines used in the present study were human recombinant proteins, implying that their actions on elephant chondrocytes may not represent the actions of species-specific cytokines. Nevertheless, the significant enhancement of *MMP3* and *MMP13* expression achieved by the combination of OSM and IL-1β provides important information regarding the action of these cytokines in the catabolic processes of elephant OA, which are similar to OA pathogenesis in other animals [17, 39].

Enzymes MMP-3 and MMP-13 are members of a zinc-dependent group of endopeptidases and considered crucial for the destruction process of cartilage ECM that occurs in OA [7–10]. The present study reveals that the expression of elephant *MMP13* is more sensitive to induction by cytokines than *MMP3*. Among MMPs, most studies have focused on MMP-13, a collagenase-3, which is suggested to play a critical role in both the early stages and progression of OA [9, 40]. It is overexpressed in patients with OA but not in healthy patients. MMP-13 involves in cartilage degradation and also acts as a regulatory factor. It has been suggested that it plays a key role in controlling the onset of OA by leading chondrocytes from a normal to a pathological state [41]. MMP-3, stromelysin-1, is a matrix-degrading enzyme found to be increased in the serum and plasma of humans with OA, although its levels are not directly associated with OA severity [42]. Immunohistochemical assay of the synovium tissue of OA shows a high expression of MMP-3, which is positively correlated to the severity of the disease [10].

Likewise, in this study, the high expression of these enzymes in elephant chondrocytes was demonstrated under activation by the proinflammatory cytokines responsible for OA pathogenesis. Our results suggest that these enzymes, especially MMP-13, which exerts a strong response to cytokine activation, may be one of the key catabolic enzymes involved in elephant cartilage degeneration. Cytokine-induced upregulation of *MMP13* mRNA levels was accompanied by an increase of MMP-13 protein levels in the culture media. This protein was successfully measured by a test kit designed to determine the level of human MMP-13, suggesting that the structures of elephant and human MMP-13 is closely related. However, another test kit designed to analyze human MMP-3 levels could not successfully be applied to measure the level of MMP-3 protein in elephant chondrocytes. Therefore, we postulate that the MMP-3 protein structure similarity between humans and elephants falls below the threshold of the recognizable capability of the human MMP-3 monoclonal antibody provided with the test kit.

Currently, scientific evidence on OA pathogenesis in elephants is limited. Expanding information regarding the biomechanisms of the disease as well as the effectiveness of drugs will support the development of therapeutic interventions that may be helpful to treat elephant OA. As such, the present study selected four drugs commonly prescribed to treat OA in humans and other animals, namely, dexamethasone, indomethacin, etoricoxib, and diacerein. Dexamethasone is a synthetic corticosteroid previously shown to inhibit the expression of *MMP3* and *MMP13* in IL-1α-induced bovine chondrocytes and suppress cytokine-induced inhibition of matrix biosynthesis in bovine cartilage [26]. NSAIDs are generally used to reduce pain and inflammation in arthritis through inhibition of cyclooxygenase (COX) [43]. Indomethacin is a non-selective inhibitor, whereas etoricoxib is in the COX2 selective class of NSAIDs. The former has been reported to reduce the expression of *MMP1* and *MMP3* in IL-1α-induced bovine chondrocytes [23], whereas the latter has been found to decrease the levels of MMP-2 and MMP-9 [25]. Diacerein, a DMOADs, has been reported to decrease the production of IL-1-converting enzyme and IL-1β in human osteoarthritic cartilage [44] as well as suppress the expression of *MMP1*, *MMP3*, *MMP13, ADAMTS-4*, and *ADAMTS-5* in IL-1β-induced bovine chondrocytes [24]. Our results show that these drugs effectively suppress the expression of *MMP3* and *MMP13* induced by the combination of IL-1β and OSM or LPS, suggesting that they exhibited an anti-arthritic potential in the elephant chondrocytes culture model.

Moreover, this study demonstrates the protective effect of natural compounds previously reported to have anti-arthritic properties such as sesamin, andrographolide, and vanillylacetone against cytokine-induced expression of *MMP3* and *MMP13* in elephants, suggesting similarities in human and elephant OA pathogenesis, which is ameliorated by the action of these natural compounds. The concentration ranges of the natural compounds used in this study did not cause cell mortality but still effectively reduced the expression of *MMP3* and *MMP13* and were selected based on the results of the MTT cytotoxic assay [see Additional file 1]. However, the therapeutic dose of these agents on human or animal arthritis remains unclear. Therefore, the application of these agents to human or animal arthritis must be further investigated to achieve the maximum therapeutic effect.

It was reported that supplementation of sesame seed in patients with knee OA at a dose of 40 g daily for 2 months, along with standard medical therapy, improved the disease activity by reducing serum IL-6 [45]. In papain-induced rat OA, intra-articular injection of 20 μL of 1 or 10 μM sesamin reduced cartilage distortion [28]. This compound is the most prominent lignan in sesame seed oil [46] and has been reported to exert anti-arthritic effects by reducing IL-1β-induced production of proinflammatory mediators and cartilage-degrading enzymes MMP-1, MMP-3, and MMP-13, in human osteoarthritic chondrocytes via suppressing phosphorylation of NF-κB p65 and IκB and activation of the Nrf2 signaling pathway [28, 47].

Vanillylacetone, also called zingerone, is the major component of ginger root and has known antioxidant and anti-inflammatory properties [48]. In cytokine-induced degradation of porcine cartilage explant, this compound decreased the release of MMP-13 and cartilage matrix biomolecules into the culture media by suppressing the p38 and JNK MAPK signaling pathways [30]. Patients receiving one ginger extract capsule prepared from 2500 to 4000 mg of dried ginger rhizomes twice daily for 6 weeks showed a significant reduction of OA symptoms [49]. However, reports on the usage of vanillylacetone for anti-arthritic purposes in humans or animals are still limited.

Andrographolide is a major bioactive compound of *Andrographis paniculata* (Burm.f.) that was found to inhibit the expression of MMPs and inducible nitric oxide synthase in an IL-1β-induced OA model [29]. This agent reduced the productions of proinflammatory cytokines in vitro by suppressing the p38 MAPK and ERK1/2 pathways and alleviated arthritis severity in mice treated by oral administration of andrographolide 100 mg/kg/d [50]. It was reported that a combined administration of andrographolide 50 mg/kg/d and methotrexate 2 mg/kg/week in rat arthritis induced by complete Freund’s adjuvant significantly attenuated inflammatory symptoms and reduced liver injury caused by methotrexate [51]. Andrographolide has been proposed as a new potential anti-arthritic agent [52]. Therefore, it is worth further investigating the optimal dose of this agent for arthritis treatments in animals or humans. LPS are known to induce infectious arthritis and contribute to low-grade inflammation in OA pathogenesis [19, 53, 54]. They enhance the production of MMP-1, MMP-3, MMP-13, nitric oxide, and prostaglandin E2 in OA patients, leading to an increase in the area of cartilage destruction [55]. Likewise, the present study on elephant chondrocytes demonstrated a strong inducing effect of bacterial LPS on the expression of proinflammatory cytokine genes, including *IL1B*, *TNFA*, and *IL6*, together with matrix-degrading enzymes MMP3 and MMP13. These results shed light on the in vitro mechanisms of septic arthritis in an elephant chondrocyte culture model, which, when induced by LPS, showed an increased expression of proinflammatory cytokines and matrix-degrading enzymes. These effects were mitigated by dexamethasone, indomethacin, etoricoxib, and diacerein. Our findings suggest that these drugs attenuate LPS-induced inflammation and catabolic factors in both elephant and human chondrocytes.

MAPK is one of the most important signaling pathways regulating OA pathogenesis [56]. It is activated by proinflammatory cytokines, including IL-1β and OSM [12, 57], with consequent upregulation of cartilage-degrading enzyme production, including that of MMP-3 and MMP-13 [56, 58]. This study investigated the mechanisms underlying elephant OA by treating elephant chondrocytes with a combination of IL-1β and OSM via a commercial test kit commonly used to detect cellular activation in human cells via the MAPK signaling pathway. The present study shows that this test kit was successful in revealing the effects of these cytokines on the activation of p38, ERK, and JNK phosphorylation within 5–10 min before the phosphorylated forms gradually weakened. Our results support the notion that signal transduction in elephants is similar to that in humans and that this test kit is applicable to elephant chondrocytes.

## Conclusions

Overall, the findings of this study provide insight into the molecular mechanisms of OA pathogenesis in ELACs, which share similarities with those occurring in humans and other animals. In addition, anti-arthritic drugs commonly used to treat OA in humans and other animals were found to ameliorate the expression of factors associated with arthritis, including proinflammatory cytokines and enzymes responsible for cartilage degeneration. The present study provides data that contribute to the development of treatments for elephants with OA and support research into arthritis in this species.

## Methods

### Preparation of primary ELACs

A stillborn elephant calf was caused by dystocia with no clinical appearance of joint disease in an elephant camp in Chiang Mai, Thailand. Cartilage samples from the femoral head of the stifle joint were aseptically collected within 6 h postmortem during the necropsy process, which was consented by the owner. Primary ELACs were isolated by overnight digestion with type II collagenase at 37 °C. The ELACs were washed with phosphate-buffered saline and grown in Dulbecco’s Modified Eagle Medium (DMEM) containing 10% v/v fetal calf serum (FCS), penicillin (100 U/mL), and streptomycin (100 μg/mL) in a humidified incubator at 37 °C with 5% CO_2_ until confluence.

### Monolayer culture and cytokine treatment of ELACs

ELACs at a 3 × 10^5^ cells/well density were grown to confluence in DMEM containing 10% FCS. The ELACs were sustained in serum-free DMEM for 24 h, after which they were treated with proinflammatory cytokines (ProSpec, Rehovot, Israel), IL-1β (2.5 ng/mL), IL-17A (5 ng/mL), and TNF-α (5 ng/mL), either alone or in combination with OSM (2 ng/mL) for 24 h or with IL-17A (5 ng/mL) for 24 h. The ELACs were also treated with various concentrations of 0.125–1 μg/mL LPS (Sigma-Aldrich, U.S.A.). After 24 h, the cells were harvested, and the expression of *MMP3* and *MMP13* was investigated by real-time RT-PCR.

### Pellet culture and cytokine treatment of ELACs

ELACs at 1 × 10^6^ were centrifuged in 15 mL conical culture tubes at 1500 rpm for 5 min. The pellets that formed at the bottom of the tube were cultured for seven days in 500 μl of chondrogenic medium (DMEM containing 10% FCS, 1X Insulin-Transferrin-Selenium [59], 25 μg/mL ascorbic acid-2 phosphates, 10^− 7^ M dexamethasone) in a humidified incubator at 37 °C and 5% CO_2_ to allow for spherical shape formation of each pellet. The pellets were then further treated with IL-1β (5 ng/mL) and TNF-α (10 ng/mL), alone or in combination with OSM (4 ng/mL), for 3 days before being harvested for *MMP3* and *MMP13* mRNA expression analysis by real-time RT-PCR.

### Treatment with drugs and natural compounds

ELACs in monolayer cultures were treated with a combination of 2.5 ng/mL IL-1β and 2 ng/mL OSM or 0.5 μg/mL LPS for 2 h [60]. Following this, they were treated with drugs, including diacerein (2.5–10 μM; TRB Chemidica, Italy), dexamethasone (5–20 nM; Sigma-Aldrich, U.S.A.), indomethacin (2.5–10 μM; Sigma-Aldrich, U.S.A.), and etoricoxib (2.5–10 μM; Zuelling, Philippines) or with natural bioactive compounds (Sigma-Aldrich, U.S.A.), including sesamin (0.25–1 μM), andrographolide (1.25–5 μM), and vanillylacetone (20–80 μM), for 24 h. The cells were then harvested to investigate the expression of *MMP3* and *MMP13* by real-time RT-PCR, and the culture media were analyzed for protein levels of MMP-3 and MMP-13.

### Real-time RT-PCR

Total RNA was extracted from the ELACs obtained from the monolayer or pellet cultures using the Illustra RNAspin Mini RNA Isolation Kit (GE Healthcare Life Sciences, U.K.), according to the manufacturer’s protocol. The total RNA of the monolayer (0.5 μg) and pellet (0.25 μg) cultures was reverse transcribed into complementary DNA using the ReverTra Ace® qPCR RT Master Mix (TOYOBO, Japan). The elephant primer sequences were designed based on the NCBI Primer-BLAST tool in association with GenBank accession numbers and synthesized by Bio Basic, Canada (Table 1). Real-time RT-PCR was performed using the SensiFAST™ SYBR No-ROX Kit (Bioline, U.K.). Gene expression quantification was based on the 2^−∆∆Ct^ method against the expression of the glyceraldehydes-3-phosphate dehydrogenase gene (*GAPDH*) as a housekeeping gene [61].

**Table 1:** Real-time RT-PCR primer sequences

Gene	Primer sequence (5′-3′)
*MMP3*	Forward: AAAGGCAGGCATTTTTGGCG Reverse: AGGGTGAGGGTAGCTCTCG
*MMP13*	Forward: AGTTCCAAAGGCTACAACTT Reverse: CGCCAGAAGAATCTGTCTTT
*IL1β*	Forward: CTTGGTGCTTTCTGGTCCTTAT Reverse: AGACAAATCGCTTTTCCATCCT
*IL6*	Forward: GGCACTGGCAGGAAACAATC Reverse: GCATTTGCAGTTGGGTCAGG
*TNFα*	Forward: ATCAGCCGTATCGCTGTCTC Reverse: CCAAAGTAGACCTGCCCAGA
*GAPDH*	Forward: ATCACTGCCACCCAGAAGA Reverse: TTTCTCCAGGCGGCAGGTCAG

### Measurement of MMP-3 and MMP-13 levels in the culture media

The levels of MMP-3 and MMP-13 enzymes in the culture media were measured using human MMP-3 (catalog number: E-CL-H0931) and MMP-13 (catalog number: E-CL-H0127) sandwich ELISA kits (Elabscience, China), according to the manufacturer’s instructions. Briefly, 100 μl of MMP-3 or MMP-13 standard and sample (culture media) was added to the monoclonal antibody against the proteins (MMP-3 or MMP-13) pre-coated micro CLIA plate well, then incubated at 37 °C. After 90 min of incubation, the standard and sample were discarded, and 100 μl of a biotinylated detection antibody working solution was added to each well. The plate was incubated for 1 h at 37 °C, followed by three washings. A horseradish peroxidase conjugate (HRP) working solution was then added to each well (100 μl/well) and left to incubate at 37 °C for 30 min. After washing, 100 μl of substrate mixture solution was added to each well before being incubated in the dark for 5 min at 37 °C. The luminescence value was detected using a Synergy H4 hybrid multi-mode microplate reader (BioTek, U.S.A.), and the protein concentrations were calculated by comparing the samples with standard curves.

### Western blot analysis of intracellular signaling molecules

ELACs were treated with a combination of the cytokines IL-1β (2.5 ng/mL) and OSM (2.5 ng/mL) at various time points. To investigate the activation of the MAPK pathway, the cells were collected in a radioimmunoprecipitation assay buffer. The cell lysates were vortexed every few minutes before centrifugation at 14,000 g for 10 min at 4 °C, after which the supernatants of the cell lysate were transferred into new tubes. The cells were lysed with a sample buffer containing 5% mercaptoethanol. Equal amounts (25 μg protein) of the cell lysates were heated for 10 min at 95 °C then subjected to 13% SDS-PAGE and transferred to a nitrocellulose membrane. After blocking non-specific proteins with 5% skim milk in TBS containing 0.1% Tween 20 (TBS-T) for 1 h, the membranes were washed with TBS-T and probed with primary antibodies (Cell Signaling Technology, U.S.A.), including rabbit anti-phosphorylated-p38 MAPK antibody, rabbit anti-phosphorylated-p44/42 MAPK antibody, rabbit anti-phosphorylated-SAPK/JNK antibody, rabbit anti-p38 MAPK antibody, rabbit anti-p44/42 MAPK antibody, rabbit anti-SAPK/JNK antibody, and mouse anti-β-actin (Biolegend, CA), at 4 °C overnight. After being washed with TBS-T, the membranes were incubated for 1 h with the secondary antibody conjugated with HRP anti-rabbit IgG or anti-mouse IgG at room temperature. The positive bands were visualized by enhanced chemiluminescence using the ChemiDoc system (Bio-Rad, U.S.A.). The intensity of the immuno-positive bands was calculated using the TotalLab TL120 software.

### Statistical analysis

The results are presented as the mean ± standard error of the mean of three independent experiments. The statistical analysis was performed using one-way analysis of variance followed by LSD for post-hoc multiple comparisons. A level of *p* < 0.05 was considered statistically significant.

## Supplementary information

**Additional file 1.** The effect of natural compounds on elephant articular chondrocytes viability by using MTT assay.

## Data Availability

The datasets used and/or analyzed during the current study are available from the corresponding author on reasonable request.

## Abbreviations

ELACs: Elephant articular chondrocytes
FCS: Fetal calf serum
IL-17A: Interleukin-17A
IL-1β: Interleukin-1beta
LPS: Lipopolysaccharides
MAPK: the mitogen-activated protein kinase
MMP: Matrix metalloproteinase
NSAIDs: Non-steroidal anti-inflammatory drugs
OA: Osteoarthritis
OSM: Oncostatin M
TNF-α: Tumor necrosis factor-alpha
