# Supplementary material for: Proinflammatory cytokines and lipopolysaccharides up regulate MMP-3 and MMP-13 production in Asian elephant (Elephas maximus) chondrocytes: attenuation by anti-arthritic agents
Source: BMC Vet Res. 2019 Nov 21;15:419. doi: 10.1186/s12917-019-2170-8 (PMC6873576; doi:10.1186/s12917-019-2170-8)
Supplement: Supplementary file 1 — Additional file 1. The effect of natural compounds on elephant articular chondrocytes viability by using MTT assay. [file 12917_2019_2170_MOESM1_ESM.pdf]

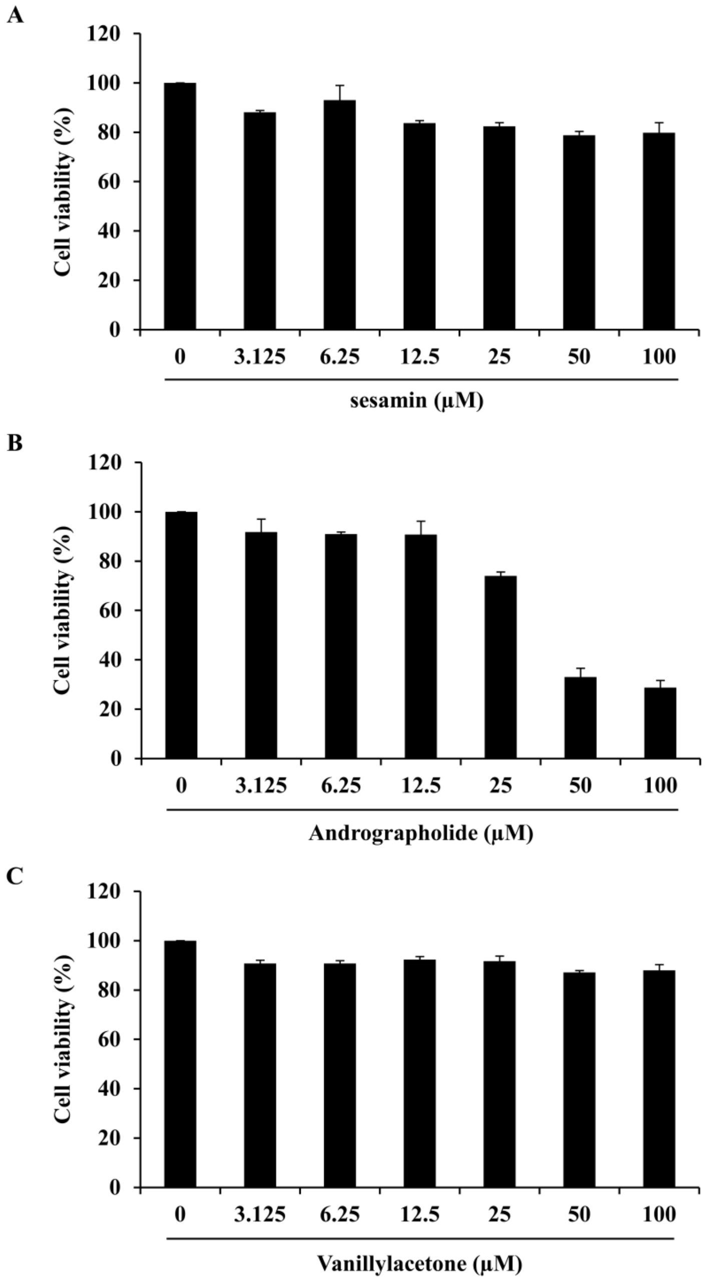

The effect of natural compounds on elephant articular chondrocytes viability.

The elephant articular chondrocytes were incubated for 24 hours with sesamin (0-100 µM) (A), andrographolide (0-100 µM) (B), and vanillylacetone (0-100 µM) (C). The viability of the cells was determined using the MTT assay. The cell viability was expressed as a percentage relative to that of 0 µM. The bar graphs were expressed as the mean ± SEM of three independent experiments.
